# Valor predictivo de proteína C reactiva al ingreso sanatorial y requerimiento de asistencia respiratoria mecánica en adultos internados por COVID-19

**DOI:** 10.31053/1853.0605.v81.n1.41799

**Published:** 2024-03-27

**Authors:** Betiana Alejandra Guidetto, Sebastian Fonseca, Alejandro Martin Abrate, Maria Teresa Politi

**Affiliations:** 1 Sanatorio Anchorena San Martin; 2 Trinidad Palermo; 3 Fisiología y de Farmacología. Facultad de Ciencias Médicas. Universidad de Buenos Aires

**Keywords:** covid-19, proteína c-reactiva, ventilación mecánica, covid-19, c-reactive protein, respiration artificial, covid-19, proteína c-reativa, respiração artificial

## Abstract

**Introducción:**

Durante la pandemia por COVID-19, los pacientes con peor evolución presentaron deterioro clínico a los 7-10 días del inicio de síntomas, lo cual sugiere que la respuesta inflamatoria podría participar de la fisiopatogenia de la enfermedad. El objetivo de este estudio fue evaluar la asociación entre los valores de proteína C reactiva (PCr) en plasma al ingreso sanatorial en adultos con COVID-19 y el requerimiento de asistencia respiratoria mecánica (ARM) durante la internación.

**Métodos:**

Cohorte retrospectiva, observacional, en un centro privado de la provincia de Buenos Aires. Se incluyeron a adultos internados con diagnóstico de COVID-19 por hisopado nasal, mediante real time transcription polymerasa chain reaction o antígeno. El desenlace primario fue la asociación entre valores altos de PCr en plasma al ingreso sanatorial (≥8 mg/L) y el requerimiento de ARM durante la internación.

**Resultados:**

De los 1.242 pacientes enrolados, 19,4% requirieron ARM y 11,7% fallecieron durante la internación. La PCr de los pacientes que requirieron ARM fue mayor que la de los que no la requirieron (9,45 [5,20-18,70] mg/L vs 4,95 [1,80-10,70] mg/L; p < 0,01). La PCr analizada como variable continua (OR = 1,39; IC95% 1,21-1,60; p < 0,001) y como variable categórica (≥8 mg/L) (OR = 2,66; IC95% 2,19-3,78; p < 0,001) presentó una asociación significativa con el requerimiento de ARM durante la internación. Secundariamente, se encontró una asociación significativa entre PCr y mortalidad intrahospitalaria.

**Conclusión:**

El valor de PCr en plasma al ingreso sanatorial podría predecir la evolución clínica en pacientes adultos internados por COVID-19.

## Abstract (en)

**Introduction:**

During the COVID-19 pandemic, patients with worse evolution presented clinical deterioration 7-10 days after the onset of symptoms, which suggests that the inflammatory response could participate in the pathophysiology of the disease. The objective of this study was to evaluate the association between plasma C-reactive protein (PCr) on hospital admission and mechanical ventilation requirement during hospitalization in adults with COVID-19.

**Methods:**

Retrospective, observational cohort at a private center in the province of Buenos Aires. Hospitalized adults diagnosed with COVID-19 by nasal swab using real time transcription polymerase chain reaction or antigen were included. The primary outcome was the association between high plasma PCr values on hospital admission (≥8 mg/L) and mechanical ventilation requirement during hospitalization.

**Results:**

Of the 1,242 patients enrolled, 19.4% required mechanical ventilation and 11.7% died during the hospitalization. The PCr of the patients who required mechanical ventilation was higher than that of those who did not require mechanical ventilation (9.45 [5.20-18.70] mg/L vs 4.95 [1.80-10.70] mg/L; p < 0.01). PCr analyzed as a continuous variable (OR = 1.39; 95%CI 1.21-1.60; p < 0.001) and as a categorical variable (≥8 mg/L) (OR = 2.66; 95%CI 2.19 -3.78, p < 0.001) presented a significant association with the requirement of mechanical ventilation during hospitalization. Additionally, a significant association was found between PCr and in-hospital mortality.

**Conclusion:**

Plasma PCr on hospital admission could predict clinical evolution in adult patients hospitalized for COVID-19.

## Resumo

**Introdução:**

Durante a pandemia de COVID-19, os pacientes apresentaram uma piora clínica de 7-10 dias após o início dos sintomas, o que sugere que a resposta inflamatória poderia participar da fisiopatologia da doença. O objetivo deste estudo foi avaliar a associação entre os valores de proteína C reativa (PCr) plasmática na admissão hospitalar em adultos com COVID-19 e a necessidade de ventilação mecânica durante a internação.

**Métodos:**

Corte observacional retrospectiva em um centro privado na província de Buenos Aires. Foram incluídos adultos hospitalizados com diagnóstico de COVID-19 por swab nasal, usando reação em cadeia da polimerase com transcrição em tempo real ou antígeno. O desfecho primário foi a associação entre altos valores plasmáticos de PCr na admissão hospitalar (≥8 mg/L) e a necessidade de ventilação mecânica durante a internação.

**Resultados:**

Os 1.242 pacientes inscritos, 19,4% necessitaram de MRA e 11,7% morreram durante a internação. A PCr dos pacientes que necessitaram de ventilação mecânica foi maior do que a daqueles que não necessitaram (9,45 [5,20-18,70] mg/L vs 4,95 [1,80-10,70] mg/L; p < 0,01). PCr analisa como variável continua (OR = 1,39; IC 95% 1,21-1,60; p < 0,001) e como variável categórica (≥8 mg/L) (OR = 2,66; IC 95% 2,19 -3,78, p < 0,001) apresentou associação significativa com a necessidade de ventilação mecânica durante a internação. Secundariamente, foi encontrada associação significativa entre PCR e mortalidade intra-hospitalar.

**Conclusão:**

O valor plasmático de PCr na admissão hospitalar pode predizer a evolução clínica em pacientes adultos internados por COVID-19.

**CONCEPTOS CLAVE**

**Qué se sabe sobre el tema**

El deterioro clínico en pacientes con COVID-19 está dado, al menos en parte, por una respuesta inflamatoria desmedida. Los marcadores inflamatorios plasmáticos podrían anticipar la evolución clínica de pacientes con COVID-19. Aún no existe información suficiente para respaldar el uso sistemático de marcadores inflamatorios plasmáticos en la toma de decisiones clínicas en pacientes con COVID-19.

**Qué aporta este trabajo**

En adultos internados por COVID-19, los valores de proteína C reactiva en plasma al ingreso sanatorial pueden predecir el requerimiento de asistencia respiratoria mecánica. Posiblemente, estos valores también puedan predecir la mortalidad intrahospitalaria. El valor de corte de proteína C reactiva que maximiza la sensibilidad y la especificidad para predecir el requerimiento de asistencia respiratoria mecánica es 7,5 mg/L. Este estudio aporta información valiosa de pacientes con COVID-19 de Latinoamérica, la cual aún es escasa.

**Divulgación**

El siguiente trabajo analiza la relación entre los valores de proteína C reactiva (PCr) (una proteína producida por el hígado ante procesos inflamatorios o infecciosos) en sangre al ingreso sanatorial y su relación con el requerimiento de asistencia respiratoria mecánica (ARM) en pacientes internados por COVID-19 en un sanatorio de la provincia de Buenos Aires. De forma secundaria, se analiza la relación entre estos valores y la mortalidad durante la internación y también el comportamiento de otros parámetros inflamatorios (tal como ferritina y eritrosedimentación). De acuerdo a nuestro análisis, la PCr podría ser predictora de mala evolución y requerimiento de ARM en estos pacientes, por lo que podría considerarse en su seguimiento durante la internación.

## Introducción

A finales del 2019 el nuevo coronavirus denominado SARS-CoV-2, causante de COVID-19, emergió como problemática de Salud Pública, propagándose rápidamente a nivel mundial y generando una nueva pandemia ^1^ ^2^ . Las manifestaciones clínicas se presentaron en su mayoría como cuadros de vía aérea superior leves con buena evolución, pero en algunos pacientes generó cuadros graves, principalmente neumonía, que incluyeron la muerte ^3^ . Si bien no se conoce con exactitud la fisiopatología de esta enfermedad, el deterioro clínico en pacientes con COVID-19 suele presentarse a los 7-10 días del inicio de síntomas, momento en el cual la carga viral desciende, lo que podría sugerir que la patología está dada, al menos en parte, por una respuesta inflamatoria desmedida ^4^ . Distintos estudios evidenciaron que en pacientes con presentaciones severas existe un estado inflamatorio manifestado por un aumento de dichos parámetros, entre ellos la proteína C reactiva (PCr), junto con una respuesta inmune exacerbada ^5^ ^6^ . Por otro lado, sabemos que la PCr es sintetizada por el hígado y eleva sus niveles ante una respuesta inflamatoria, mayormente en infecciones bacterianas o virales, donde actúa uniéndose a estos patógenos y desencadenando su eliminación por las células fagocíticas ^7^ ^8^ .

Actualmente, existe información disponible que sugiere que los valores de PCr podrían anticipar la evolución clínica de pacientes con COVID-19. Algunos estudios, tal como los de G. Wang *et al.* ^8^ y X. Luo *et al.* ^7^ , evidenciaron que los pacientes que iniciaron la presentación clínica de esta enfermedad con un cuadro no severo, pero con valores de PCr francamente elevados, tuvieron una peor evolución clínica que aquellos que iniciaron el cuadro con valores de PCr normales o moderadamente elevados. Así también, un estudio publicado por I. Prasetya *et al.* estableció que el aumento de marcadores inflamatorios -incluyendo la PCr- se asociaba a enfermedad severa por COVID-19 ^9^ . En su conjunto, esta evidencia sugiere que esta proteína podría ser un predictor de mala evolución clínica en estos pacientes. Sin embargo, aún no existe información epidemiológica suficiente para respaldar el uso sistemático de PCr en plasma en la toma de decisiones clínicas en pacientes con COVID-19. Adicionalmente, la información disponible en Latinoamérica acerca sobre este tema es limitada. Por ejemplo, un estudio pequeño realizado en Perú exploró distintos valores de corte de PCr para predecir una evolución grave en adultos internados por COVID ^10^ .

El objetivo primario de este estudio fue analizar la asociación entre PCr en plasma al ingreso sanatorial de pacientes que cursaron internación por COVID-19 y el requerimiento de asistencia respiratoria mecánica (ARM). De forma secundaria se evaluó la asociación entre PCr en plasma al ingreso sanatorial y muerte intrahospitalaria. Asimismo, se propuso describir la evolución temporal de la PCr a lo largo de la internación.

## Materiales y Métodos

### Diseño y población

Se realizó un estudio de cohorte retrospectiva en un centro único de la provincia de Buenos Aires a partir de una base de datos de pacientes internados por COVID-19. Los criterios de inclusión fueron: 1) encontrarse internado con diagnóstico de COVID-19 como motivo primario de internación, confirmado por real time transcription polymerasa chain reaction (RT-PCR) y/o antígeno para SARS-CoV-2 en hisopado nasofaríngeo, 2) ser mayor de 18 años de edad, 3) contar con el valor de PCr en plasma dentro de las 48 hs del ingreso sanatorial. Los criterios de exclusión fueron: 1) no contar con información respecto al requerimiento de ARM durante la internación, 2) encontrarse en ARM previo a obtener la muestra de plasma para la cuantificación de PCr y 3) encontrarse en ARM al ingreso sanatorial, al provenir derivado de otra institución.

### Datos clínicos y bioquímicos

Se recolectaron datos demográficos y clínicos de las historias clínicas digitales tal como sexo, edad, comorbilidades. Asimismo se recolectaron datos de análisis bioquímicos tales como PCr, eritrosedimentación (VSG) y ferritina (Fr) al ingreso sanatorial y durante la internación.

La confirmación del diagnóstico de COVID-19 se realizó mediante RT-PCR y/o antígeno para SARS-CoV-2 en hisopado nasofaríngeo.

La principal variable de exposición evaluada fue valores altos de PCr en plasma al ingreso sanatorial. El límite superior normal de los valores de esta proteína en plasma varía de acuerdo a la literatura entre 8 y 10 mg/L (8,9,11). El hallazgo de valores por encima de estos podría asociarse a infecciones. En nuestro estudio definimos a priori a valores ≥ 8 mg/L como un valor de PCr aumentada en plasma ya que: 1) este es el valor de corte que se utiliza en la práctica habitual en nuestro centro, 2) está de acuerdo con los valores utilizados en la literatura en relación a la evolución clínica del COVID-19 ^8^ y 3) dado que existe evidencia de que algunas infecciones virales pueden asociarse a variaciones pequeñas del valor de PCr ^9^ ^12^ ^13^ .

La variable de desenlace evaluada fue el requerimiento de ARM definida como la necesidad de utilizar asistencia respiratoria mecánica por presentar insuficiencia respiratoria y/ o desaturación que no corrige con dispositivos no invasivos, por necesidad de mejorar la ventilación y/o signos de shock ^14^ .

El punto final primario a evaluar fue la asociación entre valores altos de PCr en plasma al ingreso sanatorial (≥8 mg/L) y el requerimiento de ARM durante la internación.

### Análisis estadístico

La diferencia de PCr en plasma al ingreso sanatorial en pacientes según el requerimiento de ARM se valoró a través de un test de Wilcoxon de suma de rangos (Wilcoxon rank-sum test) (o Mann-Whitney U test).

Si bien hipotetizamos que pacientes con valores más altos de PCr en plasma al ingreso sanatorial tendrían mayor riesgo de tener presentaciones más grave de la enfermedad, para simplificar este abordaje en el desenlace primario dicotomizamos a la variable PCr según un valor definido a priori (≥8 mg/L), tal como lo han realizado previamente otros autores ^8^ . En función de verificar la robustez de esta asociación, evaluamos los valores de PCr en plasma primero considerando a la variable como continua y luego dicotomizando dicha variable en el valor de corte establecido a priori (≥8 mg/L). En ambos casos utilizando modelos de regresión logística univariados.

La contribución de otras covariables clínicas que pudieran mejorar la predicción de valores altos de PCr en plasma al ingreso sanatorial (≥8 mg/L) se valoraron mediante un modelo de regresión logística multivariado. Se evaluaron covariables con un valor de p < 0,02 en un modelo de regresión logística univariado. El modelo final se construyó de manera manual por el método de step-forward ingresando las variables en función de su p-valor, en orden creciente. Los desenlaces exploratorios se valoraron de manera descriptiva, mediante gráficos y tablas.

Estimamos que un tamaño muestral de 1.175 personas sería necesario para detectar un aumento del odds del requerimiento de ARM de OR = 1,50 (IC95% 1,40-1,60) asumiendo que la prevalencia esperada de valores aumentados de PCr al ingreso sanatorial en pacientes COVID es de 65% ^15^ y que la prevalencia esperada de requerimiento de ARM en esta población es de 20% ^9^ , con un nivel de significancia del 5% y un poder del 80% (Anexo 1). A este tamaño muestral agregamos un 5-7% adicional ante posibles pérdidas en el seguimiento durante el estudio.

Asimismo, a partir de esta prevalencia esperada de requerimiento de ARM y la recomendación de contemplar 10 eventos por cada covariable ^15^ , consideramos como estrategia conservadora la incorporación de hasta 5 covariables al modelo multivariado a partir de tamaño muestral establecido.

Todos los análisis estadísticos se realizaron a dos colas, considerando un nivel de significancia del 5% y un poder del 80%. Todos los análisis y gráficos se realizaron en STATA versión 14 y en R versión 3.6.0.

### Consideraciones éticas

Este estudio fue evaluado por la comisión de docencia e investigación del Sanatorio Anchorena de San Martín y aprobado por el comité de bioética HIGA Eva Perón San Martín. Si bien no se solicitó un consentimiento informado específico para este estudio, dada su naturaleza retrospectiva, todos los pacientes participantes al ingreso de su internación consintieron al uso de sus datos personales de forma anónima para fines científicos al ingreso sanatorial.

## Resultados

**Figura 1:**
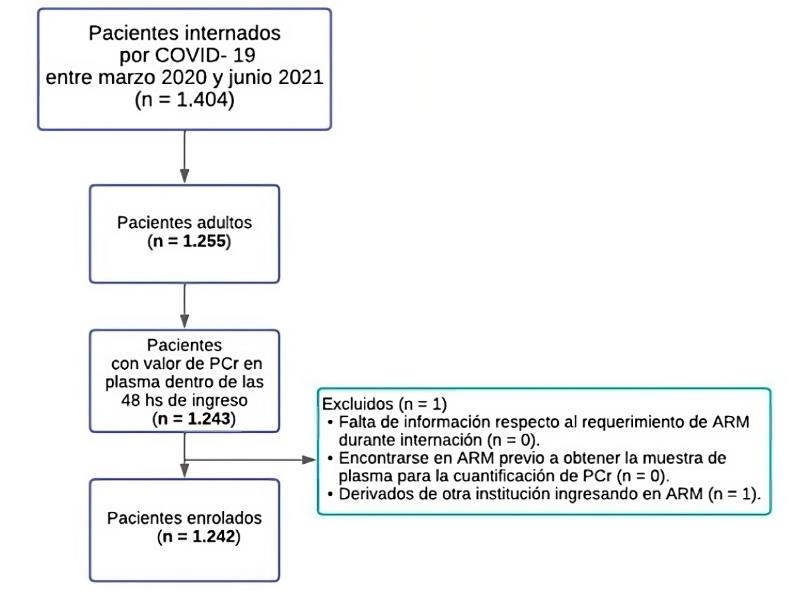
Diagrama de flujo de pacientes enrolados. **PCr:** Proteína C reactiva. ARM: Asistencia respiratoria mecánica.

Se evaluaron de forma retrospectiva a 1.404 pacientes que cursaron una internación por COVID-19 en nuestro centro entre marzo de 2020 y junio de 2021. Se incluyeron únicamente a los pacientes adultos (n = 1.255) que presentaron registros de PCr dentro de las 48 hs de internación (n = 1.243). Se excluyó a 1 paciente por haber sido derivado de otra institución en ARM. Finalmente, se enrolaron 1.242 pacientes (Figura 1).

Del total de los pacientes enrolados, la mayoría fueron hombres (57,7%) de media edad (56,3 ± 16 años). El 19,4% de los pacientes requirieron ARM y 11,7% fallecieron durante la internación.

A grandes rasgos, los pacientes con valores de PCr ≥ 8 mg/L eran mayormente hombres y de mayor edad (Tabla 1).

**Tabla 1:** Características clínicas basales de los pacientes internados por COVID-19. PCr: Proteína C reactiva. ERC: Enfermedad renal crónica. EPOC: enfermedad pulmonar obstructiva crónica. VIH: virus de inmunodeficiencia humana Variable con distribución normal expresada como media y desvío estándar (Anexo 2).

**Características clínicas**	**PCr < 8 mg/L PCr ≥ 8 mg/L**	
	(n = 769)	(n = 473)
**Edad - años⌠**	54,9 ± 16,8	58,6 ± 14,2
**Hombres - n (%)**	410 (53,3%)	307 (64,9%)
**Hipertensión arterial - n (%)**	306 (39,7%)	208 (43,9%)
**ERC- n (%)**	18 (2,3%)	17 (3,5%)
**Diabetes - n (%)**	148 (19,2%)	96 (20,3%)
**Tabaquismo - n (%)**	39 (5%)	23 (4,8%)
**Ex-tabaquismo - n (%)**	97 (12,6%)	66 (13,9%)
**Insuficiencia cardíaca - n (%)**	17 (2,2%)	12 (2,5%)
**Obesidad - n (%)**	161 (20,9%)	113 (23,8%)
**EPOC - n (%)**	33 (4,2%)	23 (4,8%)
**Enfermedad reumatológica (%)**	4 (0,5%)	6 (1,2%)
**Inmunosupresión - n (%)**	21 (2,7%)	21 (4,4%)
**VIH - n (%)**	5 (0,6%)	2 (0,4%)

Como primera aproximación, se encontró que el valor de PCr en plasma al ingreso sanatorial en los pacientes que requirieron ARM durante la internación fue mayor que en aquellos que no la requirieron (9,45 [5,20-18,70] mg/L vs 4,95 [1,80-10,70] mg/L; p < 0,01) (Anexo 3).

En el análisis univariado, se encontró una asociación significativa entre el aumento de cada 10 unidades del valor de PCr al ingreso sanatorial y el requerimiento de ARM (OR = 1,39; IC95% 1,21-1,60; p < 0,001) (Anexo 4).

Considerando un valor de corte establecido a priori de PCr ≥8 mg/L, el grupo de pacientes con PCr alta en plasma al ingreso sanatorial tuvo una mayor prevalencia de requerimiento de ARM en relación a aquellos que no presentaron valores altos de PCr en plasma al ingreso (58,3 % vs 41,7 χ = 51,9; p < 0,001), cumpliéndose los supuestos del test de chi cuadrado (Anexo 5). En un modelo de regresión logística univariado, presentar PCr alta (≥8 mg/L) en plasma al ingreso sanatorial se asoció significativamente con el requerimiento de ARM durante la internación (OR = 2,81; IC95% 2,11-3,74; p < 0,001). En un modelo de regresión logística multivariado, al agregar edad, obesidad, diabetes, hipertensión arterial, enfermedad renal crónica y tabaquismo, se mantuvo la asociación significativa entre valores de PCr altos (≥8 mg/L) en plasma al ingreso sanatorial y el requerimiento de ARM durante la internación (OR = 2,65; IC95% 1,97-3,56; p < 0,001) (Tabla 2) El sexo no presentó significancia estadística en la exploración de covariables (Anexo 6).

De manera exploratoria, se encontró una asociación significativa entre el aumento de cada 10 unidades del valor de PCr al ingreso sanatorial y la muerte intrahospitalaria (OR= 1,25; IC95% 1,08-1,43; p < 0,002). De manera similar, presentar PCr alta (≥8 mg/L) en plasma al ingreso sanatorial se asoció significativamente con la mortalidad intrahospitalaria (OR = 2,17; IC95% 1,53-3,07; p < 0,001) (Anexo 7).

**Figura 2:**
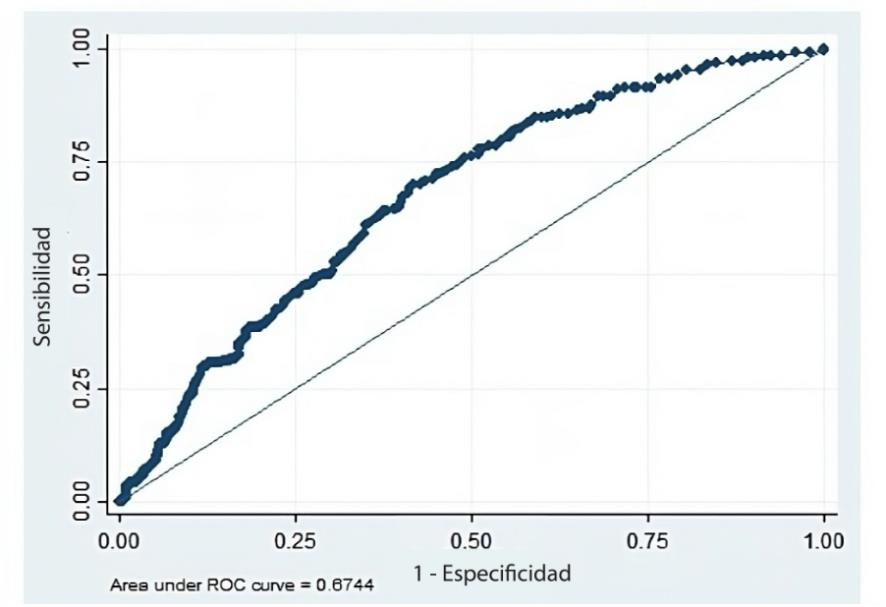
Curva ROC (receiver operating characteristic) para proteína C reactiva en plasma al ingreso sanatorial y requerimiento de asistencia respiratoria mecánica durante la internación. AUC = 0,6744.

A fin de evaluar el desempeño de distintos valores de corte de PCr en plasma al ingreso sanatorial, se realizó un análisis con una curva ROC (Figura 2). El área bajo de la curva (AUC) de dicha curva ROC fue AUC = 0,6744 (IC95% 0,64-0,71). Asimismo, encontramos que el valor de corte de PCr establecido a priori en este estudio (≥8 mg/L) tuvo una sensibilidad del 58,3% y una especificidad de 65,9%, con un 64,4% de la población correctamente clasificada, un likelihood ratio positivo de 1,7 y un likelihood ratio negativo de 0,6 (Anexo 8).

A su vez, encontramos que el rango de valores de PCr en plasma al ingreso sanatorial que presenta simultáneamente valores de sensibilidad y especificidad ≥50% se encuentra entre 5 y 9,6 mg/L. El rango de valores de PCr que presenta valores de sensibilidad y especificidad ≥60% se encuentra entre 6,7 y 7,7 mg/L. Finalmente, encontramos que el valor de corte de PCr que maximiza tanto la sensibilidad como la especificidad es 7,5 mg/L (sensibilidad 62,4% y especificidad 63,7%).

**Figura 3:**
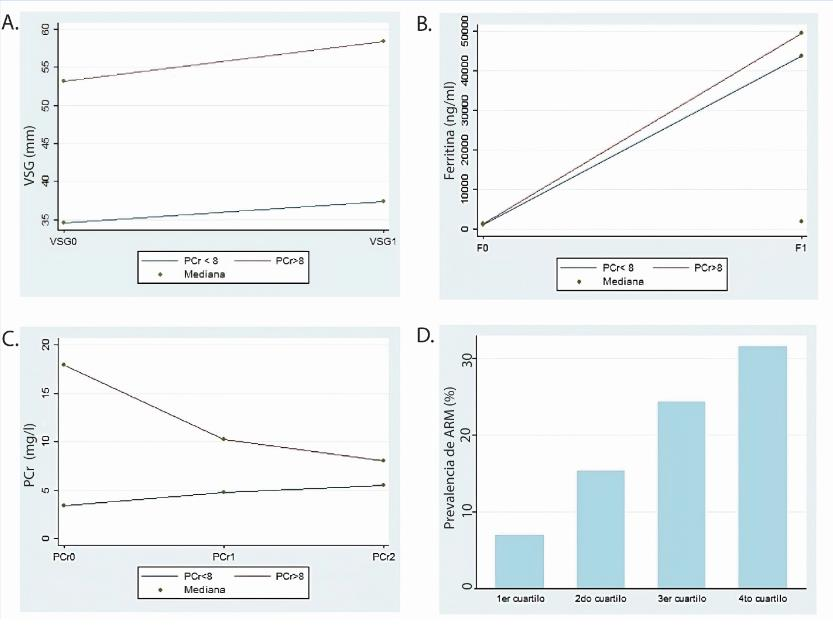
Valor de eritrosedimentación (VSG) en mm (A.), ferritina en ng/ml (B.) y proteína C reactiva (PCr) (C.) en mg/L y su evolución durante la internación, categorizado en grupos según valor de PCr en plasma al ingreso (< o ≥ 8 mg/L). VSG0: Primer valor de VSG en plasma al ingreso sanatorial. VSG1: Segundo valor de VSG en plasma durante la internación. F0: Primer valor de ferritina en plasma al ingreso sanatorial. F1: Segundo valor de ferritina en plasma durante la internación. PCr1: Segundo valor de PCr en plasma medido durante la internación. PCr2: Tercer valor de PCr en plasma medido durante la internación. D. Requerimiento de asistencia respiratoria mecánica (ARM) de acuerdo a valores de PCr en plasma al ingreso sanatorial, clasificado en cuartilos. 1er cuartilo: PCr 0-2,2 mg/L. 2do cuartilo: PCr 2,3-5,8 mg/L. 3er cuartilo: PCr 5,9-12,3 mg/L. 4to cuartilo: PCr >12,4 mg/L.

Por último, se graficaron de forma exploratoria los valores de VSG, ferritina y PCr al ingreso y su evolución durante la internación, categorizando la muestra en dos grupos de acuerdo a su valor de PCr al ingreso (< o ≥ 8 mg/L). Encontramos que el valor de VSG al ingreso en los pacientes del grupo de PCr ≥ 8 mg/L fue mayor en aquellos que del grupo PCr < 8 mg/L. Sin embargo, la tasa de crecimiento fue similar en ambos grupos (Figura 3A). En cuanto al valor de ferritina, al ingreso fue similar en ambos grupos, mientras que la tasa de crecimiento fue mayor en el grupo de PCr ≥ 8 mg/L (Figura 3B). Cuando analizamos la evolución de la PCr durante la internación encontramos que en aquellos que presentaban un valor de PCR ≥ 8 mg/L al ingreso, la misma no continuó en aumento, y se asemejan los valores al grupo de PCR < 8 mg/L (Figura 3C).

Por último, se graficó la prevalencia de ARM de acuerdo a los cuartilos de los valores de PCr en plasma al ingreso sanatorial, en el cual se evidenció que la prevalencia de ARM aumentaba en cada cuartilo de valor de PCr en plasma respecto al previo (Figura 3D).

## Discusión

En este estudio encontramos que en pacientes adultos internados por COVID-19 los valores de PCr en plasma al ingreso sanatorial, valorados tanto de manera continua como de manera categórica, pueden predecir el requerimiento de ARM. Dicha asociación se mantuvo aún luego de ajustar por variables clínicamente relevantes, tales como edad, obesidad, diabetes, hipertensión arterial y tabaquismo. Asimismo, los valores de PCr en plasma al ingreso sanatorial se encontrarían exploratoriamente asociados con mortalidad intrahospitalaria.

Estos hallazgos coinciden con la literatura disponible. En un estudio realizado en China en 209 pacientes internados por COVID-19 no severo al ingreso, G. Wang et al encontraron una asociación significativa (OR = 1,06; IC95% 1,03-1,09) entre la PCr en plasma al ingreso sanatorial y la evolución a formas severas de COVID-19 ^8^ . En el estudio realizado por Prasetya et al. en Indonesia en 391 pacientes también se reportó una asociación entre PCr en plasma y requerimiento de terapia intensiva por mala evolución de la enfermedad durante la internación (OR = 1,02; IC95% 1,02-1,03), aún luego de ajustar por covariables ^9^ .

Asimismo, estos autores documentaron una asociación estadísticamente significativa entre PCr en plasma y mortalidad (OR = 1,01; IC95% 1,01-1,02) luego de ajustar por las mismas covariables ^9^ . M. Ahnach et al. reportaron en 145 pacientes en Marruecos una asociación entre PCr en plasma y presentaciones severas de COVID-19 (OR= 1,11; IC95% 1,01-1,22) ^11^ .

En nuestro estudio encontramos un OR mayor en comparación con los trabajos mencionados, lo cual podría deberse a que medimos un punto final más duro (i.e., requerimiento de ARM) en lugar de la mala evolución clínica, tal como aparece en la literatura mencionada. Por otro lado, la población analizada en este estudio presentó una mayor prevalencia de comorbilidades (e.g., hipertensión y diabetes), en comparación con dichos estudios, lo cual podría estar indicando una mayor inflamación basal en nuestra población, sugiriendo mayor gravedad. Otra consideración que debe hacerse son las diferencias étnicas, ya que ninguno de los trabajos mencionados de la literatura fue realizado en Latinoamérica. Asimismo, nuestro estudio incluye un tamaño muestral varias veces mayor al de los estudios mencionados, pudiendo haber en ellos cierto sesgo de selección asociado a tamaños muestrales pequeños.

Un punto potencialmente controversial de nuestro estudio es el valor de corte elegido para PCr en plasma al ingreso sanatorial como variable predictora. Si bien el valor de corte de ≥8 mg/L está de acuerdo con los valores utilizados en la literatura en relación a la evolución clínica del COVID-19, otros valores se han tenido en cuenta en otras publicaciones.

Por ejemplo, Prasetya et al. encontraron una asociación significativa entre valores de corte ≥47 mg/L y progresión a enfermedad grave en pacientes con COVID-19, con una sensibilidad de 65% y una especificidad de 89%, (p < 0,001) ^9^ . M. Ahnach et al. tomaron ≥10 mg/L como valor de corte en relación a COVID-19 severo, con una sensibilidad de 86,4% y especificidad del 70,3% ^11^ . En nuestro estudio, el valor de corte de 47 mg/L presenta una sensibilidad de alrededor del 40%, por lo cual desrecomendamos su implementación como predictor de gravedad en poblaciones similares a la nuestra. El valor de corte elegido a priori en nuestro estudio (≥8 mg/L) presentó una adecuada sensibilidad y especificidad (58% y 66%); sin embargo, se evidenció que valores entre 5 y 7,5 mg/L aumentan la sensibilidad a más del 70%, manteniendo la especificidad por encima del 50%. Para verificar la robustez de nuestras conclusiones, la variable predictora de PCr en plasma al ingreso sanatorial fue explorada como variable continua tanto como variable categórica utilizando un valor de corte definido a priori, arribando en ambos casos a la misma conclusión. Posiblemente estos resultados apoyen la estrategia de implementar el uso sistemático de PCr en plasma en la toma de decisiones clínicas en pacientes con COVID-19.

La información disponible actualmente en Latinoamérica acerca del valor predictivo de PCr en plasma en pacientes con COVID-19 se limita a pocos estudios que exploran esta variable en relación a la mala evolución de la enfermedad. Un estudio realizado en Buenos Aires concluyó que un valor elevado del índice linfocito-PCr, usualmente utilizado para otras patologías, podría sugerir mayor riesgo de mortalidad en COVID-19 ^16^ . Según nuestro conocimiento, el nuestro es el primer estudio en Argentina en explorar la PCr como valor predictivo de requerimiento de ARM en pacientes con COVID-19.

A pesar de las contribuciones de este trabajo, es necesario mencionar también sus limitaciones. En primer lugar, se trata de un estudio retrospectivo realizado en un único centro, lo cual podría limitar la generalización de sus conclusiones. En segundo lugar, los valores de PCr tomados al ingreso sanatorial no se corresponden con el mismo tiempo de evolución de la infección entre paciente y paciente. Esto podría dificultar la implementación sistemática de esta información en protocolos clínicos en los cuales se utilice a la PCr como herramienta predictora de evolución de enfermedad. Por último, en nuestro estudio no se tuvieron en cuenta en los tests de hipótesis a otros parámetros inflamatorios plasmáticos analizados en otros estudios, tales como ferritina o interleuquina-6, por no ser estos estudios realizados rutina. Por este motivo, no pudo realizarse una comparación entre ellos y PCr como predictores de evolución clínica en pacientes con COVID-19 en nuestro estudio.

## Conclusión

Como conclusión, en este estudio encontramos que en pacientes adultos internados por COVID-19 la PCr en plasma al ingreso sanatorial puede predecir el requerimiento de ARM. Como perspectiva, ésta podría potencialmente constituir una herramienta útil en la toma de decisiones médicas, tanto para el monitoreo clínico como para el manejo terapéutico de los pacientes a futuro.
